# A Novel Time-Varying Spectral Filtering Algorithm for Reconstruction of Motion Artifact Corrupted Heart Rate Signals During Intense Physical Activities Using a Wearable Photoplethysmogram Sensor

**DOI:** 10.3390/s16010010

**Published:** 2015-12-23

**Authors:** Seyed M. A. Salehizadeh, Duy Dao, Jeffrey Bolkhovsky, Chae Cho, Yitzhak Mendelson, Ki H. Chon

**Affiliations:** 1 Department of Biomedical Engineering, University of Connecticut, Storrs, CT 06269, USA; dkdao2013@gmail.com (D.D.); jeffrey.bolkhovsky@uconn.edu (J.B.); chae.cho@uconn.edu (C.C.); 2 Department of Biomedical Engineering, Worcester Polytechnic Institution, Worcester, MA 01609, USA; ym@wpi.edu

**Keywords:** motion artifact, heart rate monitoring, photoplethysmogrphy, physical activities, signal processing

## Abstract

Accurate estimation of heart rates from photoplethysmogram (PPG) signals during intense physical activity is a very challenging problem. This is because strenuous and high intensity exercise can result in severe motion artifacts in PPG signals, making accurate heart rate (HR) estimation difficult. In this study we investigated a novel technique to accurately reconstruct motion-corrupted PPG signals and HR based on time-varying spectral analysis. The algorithm is called Spectral filter algorithm for Motion Artifacts and heart rate reconstruction (SpaMA). The idea is to calculate the power spectral density of both PPG and accelerometer signals for each time shift of a windowed data segment. By comparing time-varying spectra of PPG and accelerometer data, those frequency peaks resulting from motion artifacts can be distinguished from the PPG spectrum. The SpaMA approach was applied to three different datasets and four types of activities: (1) training datasets from the 2015 IEEE *Signal Process.* Cup Database recorded from 12 subjects while performing treadmill exercise from 1 km/h to 15 km/h; (2) test datasets from the 2015 IEEE *Signal Process.* Cup Database recorded from 11 subjects while performing forearm and upper arm exercise. (3) Chon Lab dataset including 10 min recordings from 10 subjects during treadmill exercise. The ECG signals from all three datasets provided the reference HRs which were used to determine the accuracy of our SpaMA algorithm. The performance of the SpaMA approach was calculated by computing the mean absolute error between the estimated HR from the PPG and the reference HR from the ECG. The average estimation errors using our method on the first, second and third datasets are 0.89, 1.93 and 1.38 beats/min respectively, while the overall error on all 33 subjects is 1.86 beats/min and the performance on only treadmill experiment datasets (22 subjects) is 1.11 beats/min. Moreover, it was found that dynamics of heart rate variability can be accurately captured using the algorithm where the mean Pearson’s correlation coefficient between the power spectral densities of the reference and the reconstructed heart rate time series was found to be 0.98. These results show that the SpaMA method has a potential for PPG-based HR monitoring in wearable devices for fitness tracking and health monitoring during intense physical activities.

## 1. Introduction

Over the last 20 years, heart rate monitors have become widely-used training aids for a variety of sports [1]. Some heart rate monitors use photoplethysmography (PPG) technology, as this allows the device to be small and wearable [2]. The sensors, consisting of infrared light-emitting diodes (LEDs) and photodetectors, offer a simple, reliable, low-cost means of monitoring pulse rates noninvasively, both at rest and during exercise [3]. This is why they have become the sensor of choice in smart watches. HR monitoring using PPG signals has many advantages compared to using traditional ECG sensors, such as simpler hardware implementation, lower cost, and no need for daily application of electrodes [4]. Fluctuations of the PPG signal are caused by changes in arterial blood volume associated with each heartbeat, where the magnitude of the fluctuations depends on the amount of blood rushing into the peripheral vascular bed, the optical absorption of the blood, skin, and tissue, and the wavelength used to illuminate the blood. The pulse oximeter signal contains not only the blood oxygen saturation and heart rate (HR) data, but also other vital physiological information [4,5,6,7]. The fluctuations of photoplethysmogram (PPG) signals contain the influences of arterial, venous, autonomic and respiratory systems on the peripheral circulation. Utilizing a pulse oximeter as a multi-purpose vital sign monitor has clinical appeal, since it is familiar to the clinician and comfortable for the patient [4]. Even simple knowledge of HR patterns would provide more useful clinical information than just HR and blood oxygenation, especially in situations in which a pulse oximeter is the sole monitor available. One major example of such benefits can be seen in a study by Chong *et al*., which show that accurate detection of atrial fibrillation can be obtained from PPG data [8].

In addition to the acquisition of HR in response to exercise, research has recently focused on obtaining heart rate variability (HRV) information from wearable sensors including devices that use PPGs [1]. Increased HRV has been associated with lower mortality rates and is affected by both age and sex [1]. During graded exercise, the majority of studies show that HRV decreases progressively up to moderate intensities, after which it stabilizes [9]. Although there are many promising and attractive features of using pulse oximeters for vital sign monitoring, currently they are mainly used on stationary patients. This is because motion artifacts (MAs) result in unreliable HR and SpO_2_ estimation [4]. Clinicians have cited motion artifacts in pulse oximetry as the most common cause of false alarms, loss of signal, and inaccurate readings [10]. During physical activities, MA contamination in PPG signals seriously interferes with HR estimation. The MAs are mainly caused by ambient light leaking into the gap between the PPG sensor surface and skin surface. Besides, the change in blood flow due to movements is another MA source [11]. In practice MAs are difficult to remove because they do not have a predefined narrow frequency band and their spectrum often overlaps that of the desired signal [12]. Consequently, development of algorithms capable of reconstructing the corrupted signal and removing artifacts is challenging.

There are a number of general techniques used for artifact detection and removal. One of the methods used to remove motion artifacts is adaptive filtering [13,14,15,16]. An adaptive filter is easy to implement and it also can be used in real-time applications, though the requirement of additional sensors to provide reference inputs is the major drawback of such methods. There are many motion and noise artifact reduction techniques based on the concept of blind source separation (BSS). The aim of BSS is to estimate a set of uncorrupted signals from a set of mixed signals which is assumed to contain both the clean and noisy sources [4]. Some of the popular BSS techniques are independent component analysis (ICA) [17], canonical correlation analysis (CCA) [18], principle component analysis (PCA) [19], and singular spectrum analysis (SSA) [4,20]. Kim and Yoo [21] suggested using a basic ICA algorithm and block interleaving to remove MA. Krishnan *et al.* [22] later proposed using frequency-domain-based ICA. However, the key assumption in ICA, namely statistical independence or uncorrelation, does not hold in PPG signals contaminated by MA [23]. Salehizadeh *et al.* [4] proposed a motion artifact removal algorithm using SSA. They used SSA to decompose the corrupted segment adjacent to the clean segment and chose the SSA components in the corrupted segment that had a similar frequency range to that of the adjunct clean components. Although they reported good performance, the method cannot be applied in scenarios where the HR and SpO_2_ are varying rapidly due to corruption and movement. Acceleration data are also shown to be helpful to remove MA. For example, Fukushima *et al.* [24] suggested a spectral subtraction technique to remove the spectrum of acceleration data from that of a PPG signal. Acceleration data can be also used to reconstruct the observation model for Kalman filtering [25] to remove MA.

Two noteworthy recently published algorithms are TROIKA and JOSS [26,27], in which sparsity-based spectrum estimation and spectral peak tracking with verification, respectively, are used to estimate and monitor heart rate during intensive physical activity. Both approaches make use of PPG and accelerometer information to obtain an accurate estimation of heart rate while running on a treadmill. TROIKA has two extra stages of signal decomposition and reconstruction using singular spectrum analysis (SSA) and it then applies temporal difference operations on the SSA-reconstructed PPG. SSA components are compared to the accelerometer signals and those components with close frequencies to the accelerometer signals are discarded and the rest are used to reconstruct the signal. In JOSS and TROIKA, spectral peak tracking with verification aims to select the spectral peaks corresponding to HR. JOSS, which has been shown to estimate HR more accurately than TROIKA, is based on the idea that the spectra of PPG signals and simultaneous acceleration signals have some common spectrum structures, and thus formulates the spectrum estimation of these signals into a joint sparse signal recovery model using the multiple measurement vector (MMV) model. MMV is used for joint spectrum estimation based on PPG and accelerometer data, which is in contrast to the single measurement vector (SMV) model that was used in TROIKA and based on only a single PPG signal. Although JOSS has been shown to be much more accurate than previous methods for reconstruction of heart rate from MA-contaminated PPG signals, the main disadvantage of the method is it can merely provide smoothed HR reconstruction estimations. Neither time-domain PPG signal reconstruction nor heart rate variability analysis can be done using JOSS or TROIKA. Recently, Temko proposed an approach to HR estimation based on Wiener Filtering and the Phase Vocoder (WFPV) [28]. This work showed that WFPV on average can perform better than the JOSS algorithm. The main idea of WFPV is to estimate motion artifacts from accelerometer signals and then use a Weiner filter to attenuate the motion components in the PPG signal. Phase vocoder is also applied to overcome the limited resolution of the Fourier transform and to refine the initial dominant frequency estimation.

In this paper, a new HR and also PPG signal reconstruction approach is presented using time-varying spectral analysis. The algorithm is called SpaMA and is comprised of five distinct stages: (1) time-varying power spectral density (PSD) calculation; (2) spectral filtering; (3) Motion Artifact detection; (4) HR reconstruction and (5) signal reconstruction. The idea is to calculate a window-segmented power spectral density of both PPG and accelerometer signals in real-time to scale each estimate of the PSD by the equivalent noise bandwidth of the window [29].

The simplest way to approach the first step, the PSD calculation, would be to employ a periodogram. However, it has the drawbacks that it is an inconsistent spectrum estimator, has high variance, and has leakage effects [29]. Thus, a dominant spectral peak can lead to an estimated spectrum that contains power in frequency bands where there should be no power. However, both problems can be solved by down-sampling the raw signal and then using a sufficiently small frequency step by setting a large number of frequency points. Thus, in this study we resample the signal from the original sampling frequency to 1/4 of it and then we apply the periodogram algorithm with frequency resolution of 0.001. Next, we limit the spectrum to the heart rate frequency range of (0.5 Hz–3 Hz) and take the frequency and power information of the first three peaks in the PSD at each window and signal segment. We are assuming that the heart rate component in a typical clean (motion free) PPG signal is always the dominant frequency component in the time-varying power spectrum, thus, the highest peak of the spectrum corresponds to the HR frequency. Thus, when movement happens the dominant component can be replaced by movement components which shift the HR to the second or third peak in the spectrum. So the third phase of the SpaMA algorithm is to compare the first three peaks and corresponding frequencies of the PPG spectrum to the first peak and frequency of the accelerometers’ spectra at each window and the idea is to choose the frequency components (out of three) that are different from the accelerometers’ frequency. We are assuming that when there is coherence between a spectral peak in the PPG and the accelerometers’ spectra this signifies a motion noise artifact in the PPG signal and that peak should be discarded in the HR reconstruction. After discarding these movement peaks in the spectrum, the next highest peak that is closest to the estimated HR of the previous window would be chosen at each window. By reconstructing the HR frequency at each window, simultaneously we can reconstruct the PPG signal by using the power, frequency and phase of the signal that corresponds to the HR frequency. That is, we reconstruct time-domain signals from the time-frequency domain. We will show in the Results section that the new SpaMA method not only provides PPG signal and HR reconstruction but also the potential to do heart rate variability analysis on the results. We will also show that SpaMA can outperform the JOSS technique in heart rate estimation by providing less error to the reference, which yields higher accuracy.

## 2. Materials and Methods 

### 2.1. Datasets

The SpaMA algorithm was evaluated on three different datasets. The first two datasets were provided for the IEEE *Signal Process.* Cup and are publically available. The three datasets are: (1) 12 PPG training datasets (running on treadmill) from an IEEE signal processing competition [30] which was initially used in [26,27]; (2) 11 PPG test datasets (e.g., arm exercise) from the IEEE signal processing competition; and (3) 10 PPG recordings from the Chon lab (running on treadmill).

(1) IEEE *Signal Process.* Competition Training Dataset: A single-channel PPG signal, a three-axis acceleration signal, and an ECG signal simultaneously recorded from 12 Asian male subjects ranging in age from 18 to 35. For each subject, the PPG signal was recorded from their wrist using a pulse oximeter (PO) with green LED (wavelength: 609 nm). The acceleration signal was also recorded from their wrist using a three-axis accelerometer. Both the PO and the accelerometer were embedded in a wristband, which was comfortably worn. The ECG signal was recorded from the chest and it is used as the reference heart rate. All signals were sampled at 125 Hz.
(2) IEEE *Signal Process.* Competition Test Dataset: The dataset consists of 11 five minute recordings which were collected from 19 to 58 years old subjects performing intensive arm movements (e.g., boxing). For each subject, PPG signals were recorded from their wrist using a pulse oximeter with green LEDs (wavelength: 515 nm). The acceleration signal was also recorded from their wrist using a three-axis accelerometer. Both the PO and the accelerometer were embedded in a wristband. An ECG signal was recorded simultaneously from their chest using wet ECG sensors. All signals were sampled at 125 Hz and sent to a nearby computer via Bluetooth.
(3) Chon Lab Dataset: This dataset was recorded in the Chon Lab from 10 healthy subjects (nine male/one female), with ages ranging from 26 to 55. For each subject, the PPG signal was recorded from their forehead using a PO (developed in our lab) with red and infrared LED (wavelength: 660 and 940 nm). The acceleration signal was also recorded from their forehead using a three-axis accelerometer. Both the pulse oximeter and the accelerometer were embedded in a headband and the signals were sampled at 80 Hz. The ECG signal was recorded as a reference from the chest using ECG sensors, sampled at 400 Hz. During data recording, subjects walked, jogged and ran on a treadmill with speeds of 3, 5 and 7 mph, respectively, for 9 min. At the end, all experimental subjects were asked to perform random arbitrary movements for 1 min.

For all three datasets, we down-sampled the data to 20 Hz since the estimation of heart rate is carried out in the frequency domain and this sampling rate is sufficiently high to obtain even heart rates that are as high as 240 beats/min or 4 Hz. Moreover, this down-sampling allows us to focus on heart rates in the lower frequencies rather than in the physiologically irrelevant higher frequency ranges. Further details of this study’s databases are given in Table 1. 

**Table 1:** PPG datasets and experiments settings.

Subject	Dataset	Activity Type	Pulse Oximeter Type	Subject’s Age/Sex
1	1 (IEEE Cup)	Type (1)	Wrist: green LED (wavelength: 609 nm)	18–38 years old; (All Male)
2				
3				
4				
5				
6				
7				
8				
9				
10				
11				
12				
13	2 (IEEE Cup)	Type (2)	Wrist: green LED (wavelength: 515 nm)	19–58 years old; (9 Male, 1 Female)
14				
15				
16		Type (3)		
17				
18				
19				
20		Type (2)		
21		Type (3)		
22				
23		Type (2)		
24	3 (Chon Lab)	Type (4)	Forehead: Red and Infrared LED (wavelength: 660 nm, 940 nm)	26–55 years old; (9 Male, 1 Female)
25				
26				
27				
28				
29				
30				
31				
32				
33				

Four types of activities were involved:

Type (1): activity involved walking or running on a treadmill for intervals of 0.5 min-1 min-1 min-1 min-1 min-0.5 min with speeds of 1–2 km/h, 6–8 km/h, 12–15 km/h, 6–8 km/h, 12–15 km/h, 1–2 km/h, respectively. The subjects were asked to purposely move the hand with the wristband to generate motion artifacts.

Type (2): activity included various forearm and upper arm exercise which are common arm motions (e.g., shaking hands, stretching, pushing objects, running, jumping, and push-ups).

Type (3): activity consisted of intensive forearm and upper arm movements (e.g., boxing).

Type (4): activity involved 1 min rest, 1 min walking (3 mph), 1 min rest, 2 min jogging (5 mph), 1 min rest, 2 min running (7 mph), 1 min rest, 1 min arbitrary movement. The ECG-based reference HR was recorded in order to assess the performance of the algorithms being tested.

In summary, the first dataset includes only Type (1), the second dataset includes both Type (1) and (2) activities, and the third dataset includes only Type (4) activities.

### 2.2. Methodology

The procedure for our new HR monitoring algorithm during intensive movements is presented in Table 2. Details of each stage will be described in subsections *i* to *v*.

**Table 2:** The proposed SpaMA algorithm: HR and PPG signal reconstruction.

Stage 1. Time-Varying Spectral analysis
1.1. Down sample the PPG and Accelerometer signal to 20 Hz.
1.2. Compute the power spectral density of both PPG and Accelerometers (0–10 Hz).
Stage 2. Spectral Filtering
2.1. Assume HR to be in the frequency range of (0.5 Hz–3 Hz), this accounts for both low and high heart rates.
2.2. The first highest three peaks and their corresponding frequencies in the PPG filtered spectrum are assumed to have HR information.
2.3. Only the largest frequency peak of the accelerometers’ spectra is used for MA detection in stage 3.
Stage 3. Motion Artifact Detection
3.1. Compare the frequencies of the three peaks in the PPG spectrum with the frequency of the largest peak in the accelerometers’ spectra. If the first or second largest peaks in the PPG spectrum are similar to that of the accelerometers’ peaks, then motion artifact is present in the PPG.
3.2. If motion artifact is detected from 3.1, then the corresponding frequency peak (usually the first or second largest peak) in the PPG spectrum should be discarded.
Stage 4. Heart Rate Tracking and Extraction from PPG Spectrum
Case (1): From 3.1—if the spectrum is corrupted by movement and only the first largest peak is corrupted, then the HR frequency should be the frequency of the second peak in the spectrum.
Case (2): From 3.1—if the spectrum is corrupted by movement and both the first and second largest peaks have similar frequencies to those of the accelerometers’ peaks, then the HR frequency should be the frequency of the third peak in the spectrum.
Case (3): Due to a gap between the pulse oximeter and a subject’s skin, the HR frequency cannot be extracted from the spectrum and in this case the previous HR frequency is used or for offline implementation a cubic spline interpolation can be applied to fill in the missing HR information.
Stage 5. PPG Signal Reconstruction
5.1. The PPG signal is reconstructed by using the amplitude, frequency and phase information corresponding to the HR components (extracted in stage 4) that are calculated from the spectrum at each window.
Heart Rate Variability Analysis
By using a sample-by-sample windowing strategy, HR can be extracted, from which dynamics of heart rate variability analysis can be obtained on the motion artifact-removed reconstructed HR time series.

#### 2.2.1. Time-Varying Spectral Analysis of PPG and Accelerometer Data

We produce a time-varying spectrum by taking a T-sec window of the signal and computing the power spectral density (PSD) of the segment and then sliding the window through the whole dataset which yields a time-frequency matrix in which each array represents the power of the signal corresponding to a specific frequency and sliding time-step (shift) of S-sec. The sliding process and frequency step specify the resolution and dimension of the time-frequency matrix. In this study, we take two different sliding window approaches depending on the application. For estimating either heart rates or heart rate variability, data are shifted sample-by-sample with no overlap for the entire dataset. This is because we are interested in capturing beat-to-beat dynamics of HRV which requires sample-to-sample estimation of PSD. Given our down-sampled data to 20 Hz in some of the database, each data point is shifted by 0.05 s. For estimating only the heart rates, we shift the data segment-by-segment rather than sample-by-sample. This coarse-grain windowing approach has less computational cost and it can provide good tracking of heart rates, but it cannot be used for HRV. The window segment length T was set to 8 s and was shifted by 2 s. We chose an 8 s data segment and a shift of 2 s because one of the goals of this work is to compare our algorithm’s results to other algorithms compared in this work (TROIKA, JOSS and WFPV) which have used this chosen data segment length and time shift [26,27]. Moreover, the assumption of 8 s data length largely stems from the fact that heart rates do not change instantaneously, hence, an 8 s duration is a reasonable choice.

**Figure 1:**
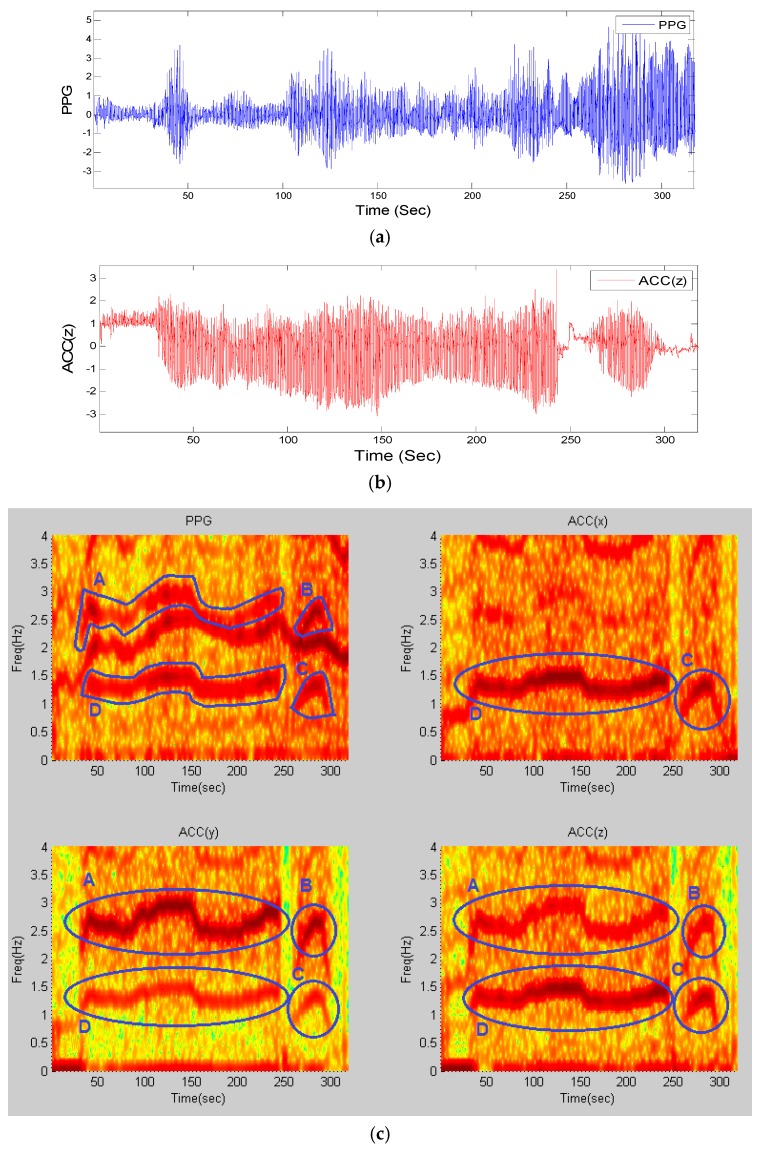
Time-frequency spectra of recording #8 from dataset (1): (**a**) PPG signal; (**b**) simultaneous Accelerometer-Z signal; (**c**) (**Top-Left**) TF spectrum of PPG; (**Top-Right**) TF spectrum of ACC(x); (**Bottom-Left**) TF spectrum of ACC(y); (**Bottom-Right**) TF spectrum of ACC(z); all computed from stage (1) of the algorithm. Blue circles and letters represent movement elements in all four spectra.

As a representative example, the resultant frequency components in the time-frequency matrix of recordings from subject #8 from the competition training dataset, for a window length of 8 s that is shifted by every 2 s, is shown in Figure 1.

The panels of Figure 1a,b show a PPG time series and the z-axis accelerometer data, respectively. From the upper left panel of Figure 1c, which represents the time-frequency plot of the PPG signal, it is observed that there are three dominant frequency components—one of them represents HR and the other two are similar to those of the accelerometers’ spectra shown in the upper right and lower left and right panels of Figure 1c. This figure illustrates four motion artifact elements A–D that are present in exactly the same areas among all spectra. By comparing the time-frequency (TF) spectrum of PPG to those of the accelerometers’ spectra, we can detect that the dynamics marked A–D in the PPG spectrum share the same frequency dynamics as those of the accelerometer spectra marked in circles. Hence, both the top and bottom marked lines in the PPG spectrum most likely represent the motion artifacts, and the unmarked frequency represents the HR. The next section details how these motion artifact frequency dynamics are detected and filtered.

#### 2.2.2. Spectral Filtering

After obtaining the power spectral density at each window, HR frequency is assumed to be confined in the range (0.5 Hz–3 Hz), which takes into account both at rest and high HR due to either tachycardia or exercise scenarios. Hence, for HR estimation, the strategy is to eliminate frequencies that are outside of this HR frequency range as they are most likely due to motion artifacts or harmonics of the HR frequency.

In general, HR frequency in the power spectral density of PPG at each window can have three different scenarios: (1) PPG is devoid of MA and there is no spatial gap between the sensor and the subject’s skin during recording; (2) PPG is corrupted by MA and there is no spatial gap between the sensor and the subject’s skin during recording and (3) there is a spatial gap between the sensor and the subject’s skin during recording. For the ideal case (1), HR can be extracted and it is most likely represented as the highest peak in the PPG spectrum; For case (2), MA dynamics can result in predominately either one or two dominant peaks depending on the severity of repetitive motions, and the HR peak is relegated to either the second or third highest peak. Non-repetitive motion artifacts will show up as a broadband spectrum without a dominant peak if they are not severe [31]. The only scenario that makes it difficult to extract HR from the spectrum is scenario (3) when there is a spatial gap between the PPG sensor and the subject’s skin during recording. In this scenario, assuming that the motion artifacts are short lasting, the missing HR values can be interpolated using the cubic spline approach.

**Figure 2:**
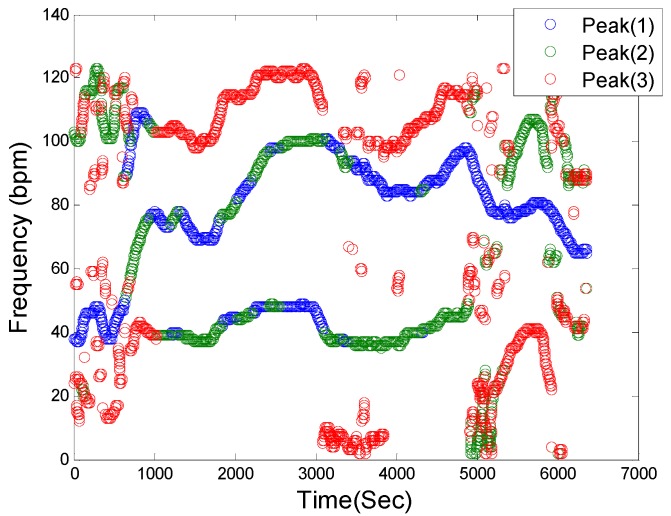
Spectral filtering. PPG time-frequency spectrum: Blue, Green and Red circles correspond to the first three highest peaks in the defined HR frequency range of (30–180 bpm), respectively, at each sliding window.

Figure 2 shows a representative filtered time-frequency spectral plot of a PPG signal. This step in the SpaMA process involves retaining only the three largest frequency peaks at each time point within the defined HR range (30–180 bpm) and they are represented as blue, green and red colors, respectively. It is our opinion that retaining only the three largest frequency peaks at each time point is reasonable for the first two cases as outlined above.

#### 2.2.3. Motion Artifact Detection

**Figure 3:**
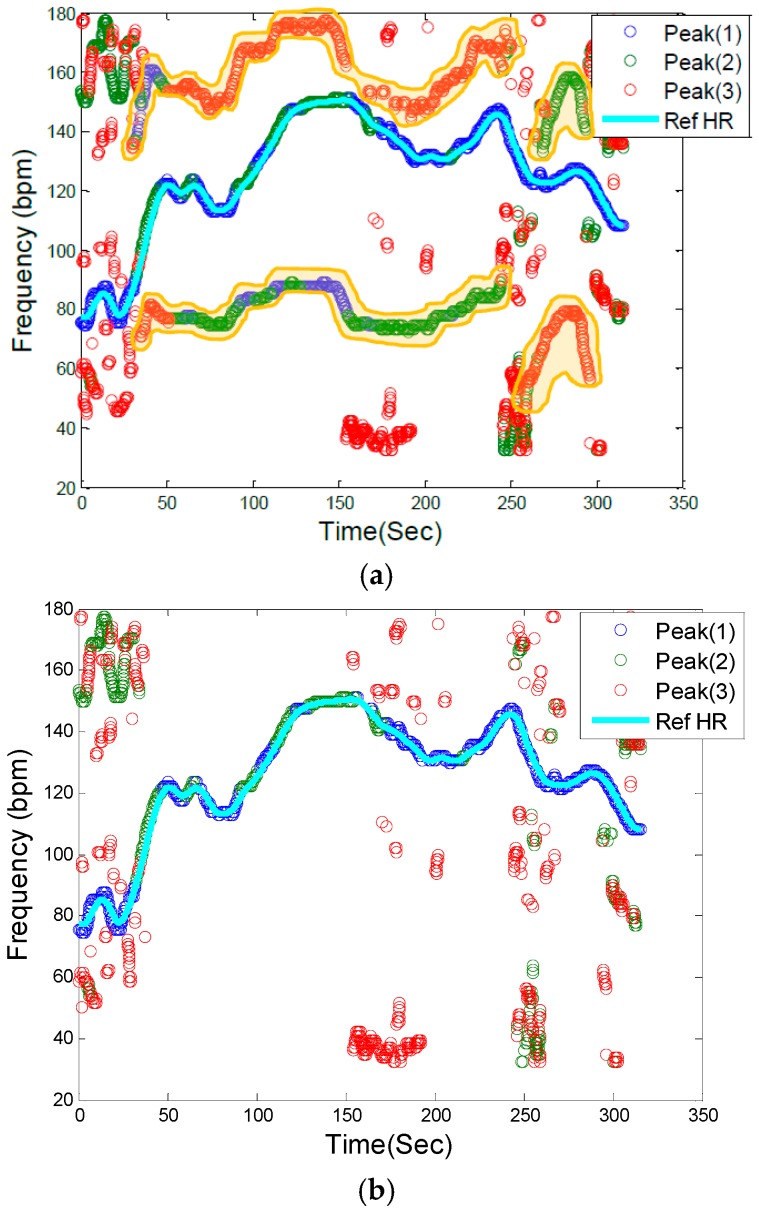
Motion artifact detection in the PPG spectrum. (**a**) Filtered PPG spectrum with movement and HR components: Shaded yellow elements (A, B, C, D) represent motion frequency components in the PPG spectrum, and the light blue line is the reference HR from clean reference ECG signal, (**b**) Filtered PPG spectrum after removing motion artifact frequency components.

Figure 3a illustrates a PPG spectrum which is identical to Figure 2, but it also identifies the frequencies associated with accelerometers, as marked by the shaded areas and the letters A–D, in the top left and two bottom panels of Figure 1c. By removing the accelerometers’ related frequencies in Figure 3a, the remaining frequency dynamics which should represent HR frequency and its harmonics are shown in Figure 3b.

#### 2.2.4. Heart Rate Tracking & Extraction

**Figure 4:**
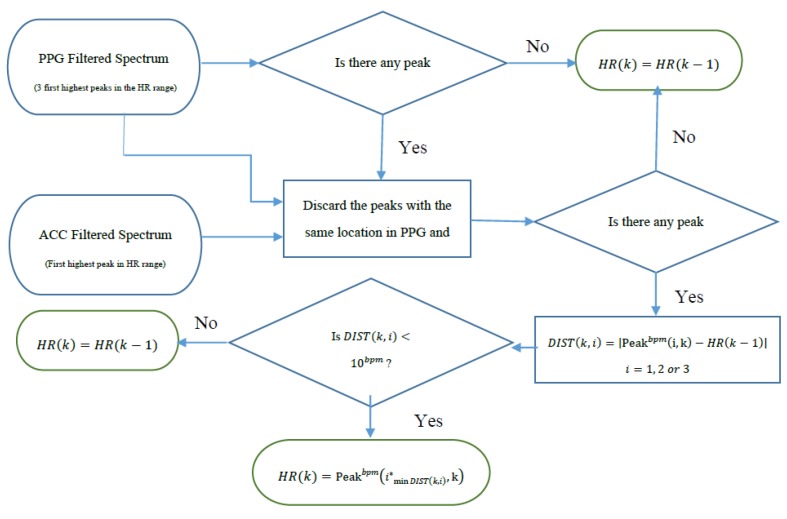
Flowchart of HR tracking and extraction.

The next step is to identify HR frequencies with time from Figure 3b. Note that in Figure 3b, there are three peaks at each time instance, thus, the question is how to identify which of the three peaks represents the HR at each time point. For the initial time window of 8 s, we require a clean data segment so that true HR can be determined. This scenario is case (1) described above in the spectral filtering section, and the detection of HR is simply the highest peak in the spectrum. The next step is to estimate HR for each sliding window of data. At this step of the algorithm, the goal is to choose a HR peak in the PPG spectrum with the knowledge of estimated HR values in previous time windows. In this step there are two main scenarios: (1) no peak exists in the spectrum that can represent HR; and (2) there is a spectral peak among the first three highest peaks of spectrum that belongs to the HR component. In case (1), where HR is not detectable in the window (e.g., due to spatial gap between the PO sensor and skin), in real-time implementation the algorithm takes the previous window’s HR value as the current HR (or simply uses the moving average of several past HR beats or some other variant), however in offline processing, a cubic spline interpolation can be used to fill in the missing HR information. In the more general case (2), where the HR peak is among the first three highest peaks in the spectrum, three possible scenarios can occur: (2-A) the windowed PPG signal is clean and the first highest peak in the spectrum represents the HR fundamental frequency; (2-B) the windowed PPG signal is corrupted by movement and at most two of the spectral peaks represent the accelerometers’ frequency components, thus the second or the third peak corresponds to HR; (2-C) while the HR spectral peak is detectable, the difference between its value and that of the previous HR is more than 10 bpm, so it will be replaced by the most recent HR value from a previous window segment (or a moving average of several past HR beats or some other variant). We set a criterion that the HR value cannot change more than 10 BPM from a previous time window. In Figure 3b, these cases are illustrated. For example, in most cases, the blue circle which represents the largest spectral peak is chosen but in other cases, either green or red circles are chosen for certain time points. For the HR peaks associated with either the green or red circles, they are chosen because either the first two highest peaks are related to accelerometers or the highest magnitude peak deviates more than 10 BPM from the previous HR value. Figure 4 summarizes the flowchart of HR tracking and extraction procedures.

**Figure 5:**
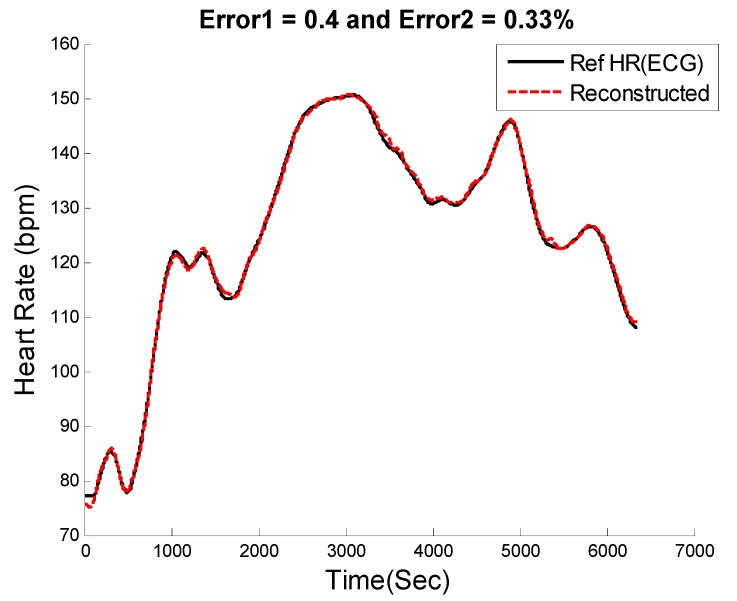
Comparison of reconstructed HR obtained from SpaMA to reference HR obtained from simultaneous ECG recordings.

Figure 5 shows the extracted HR (red color) from PPG spectra of recording #8 from the competition training dataset using our proposed approach along with the reference ECG-derived HR (black color). In order to calculate the performance of the SpaMA algorithm, the error value in each time window was calculated from the estimated HR to the reference ECG-derived HR. 

Two measurement indices of absolute error similar to the indices in [22] were used:

(1) $$Error\left(1\right)=\;\frac{1}{W}\overset{w}{\underset{k=1}{\sum }}\left|HR_{SpaMA}\left(k\right)-HR_{ref}\left(k\right)\right|$$

(2) $$Error\left(2\right)=\;\frac{1}{W}\overset{w}{\underset{k=1}{\sum }}\frac{\left|HR_{SpaMA}\left(k\right)-HR_{ref}\left(k\right)\right|}{HR_{ref}\left(k\right)}\times 100\%$$

where W is the total number of windows. 

#### 2.2.5. PPG Signal Reconstruction for HRV Analysis

**Figure 6:**
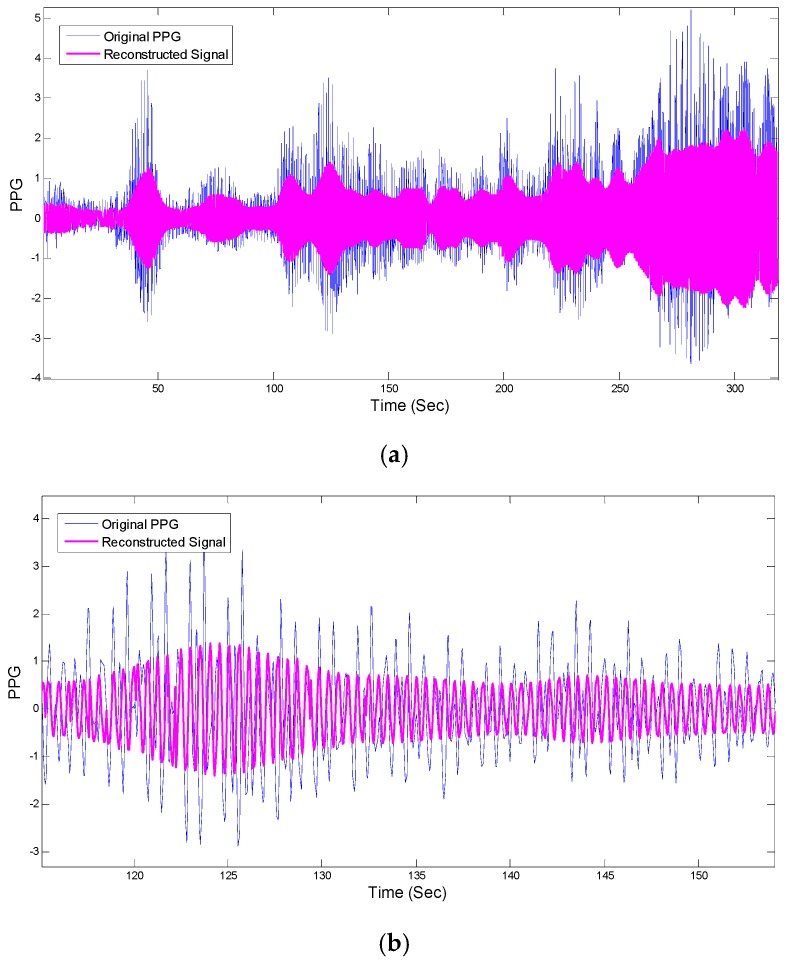
PPG signal reconstruction. (**a**) Comparison between reconstructed PPG and original recording #8 from IEEE competition training dataset; (**b**) Zoomed-in version of (**a**).

**Figure 7:**
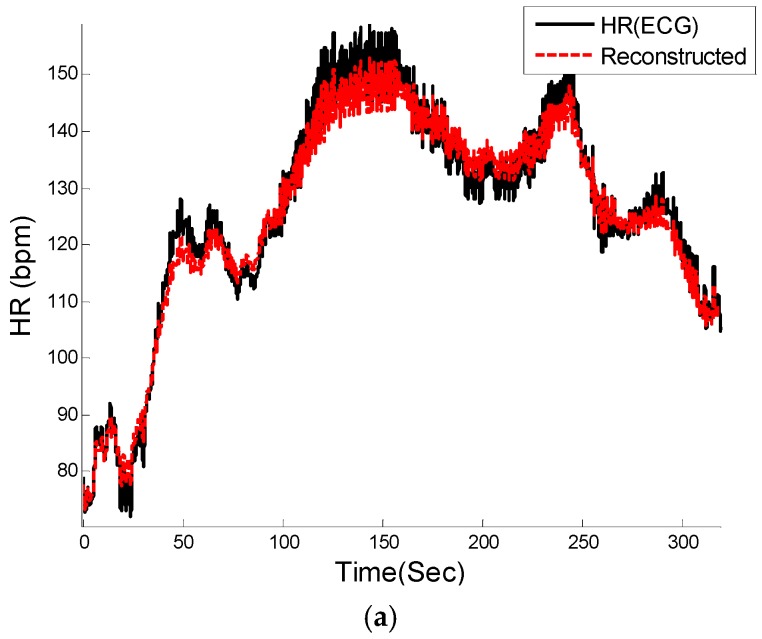

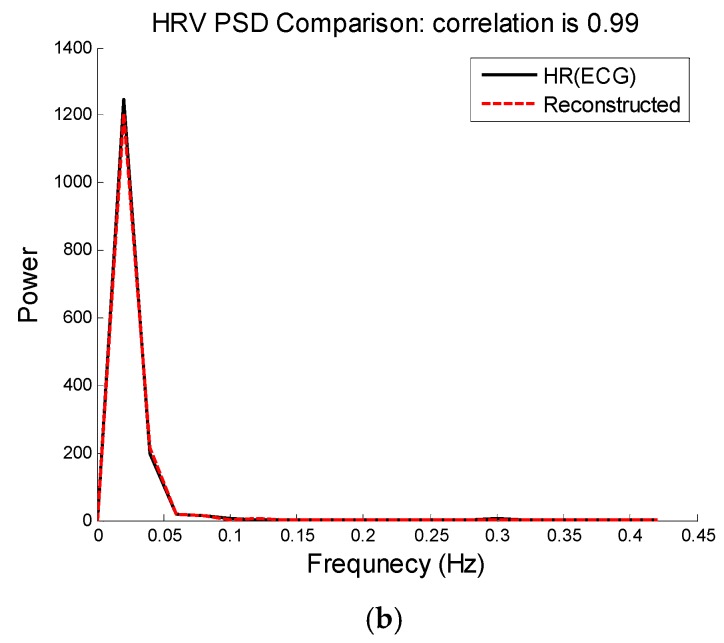
Heart rate variability analysis. (**a**) Time-domain comparison of reconstructed and reference HR; (**b**) Spectral comparison of heart rate variability between reconstructed HR and reference HR calculated from the reference ECG using Pan & Tompkins peak detection approach [32].

For HRV analysis application, the above-described procedures are identical but the only difference is the beat-by-beat shift of data rather than the 8 s data segment shift or its variant. The PPG signal can now be reconstructed using heart rate frequency, amplitude and phase changes, window-by-window using the sample-by-sample windowing process:

(3) $$Rec_{Signal}\left(k\right)=A_{HR}\left(k\right)\times \sin\left(2\pi t\left(k\right)f_{HR}\left(k\right)+\varphi_{HR}\left(k\right)\right)$$

where k=1,…, N and N is number of signal samples and total number of windows. $A_{HR}\left(k\right)$ and $\varphi_{HR}\left(k\right)$ are calculated according to the power of the signal for HR frequencies in the PSD and phase angles of complex elements in the FFT matrix that correspond to HR frequencies. The left and right panels of Figure 6 show the reconstructed PPG *versus* the original PPG and their HRV time series, respectively. Figure 7 shows comparison of HRV spectra between the reference and the reconstructed HRV time series (e.g., as shown in Figure 6) from the MA-contaminated PPG signal for dataset #8. Note that for computing HRV, we are not concerned about matching the amplitude of the reference HR, as we are interested only in the dynamics of the fluctuations in the heart rates. Given that we are able to estimate quite accurate heart rates, it is not surprising to observe similar frequency dynamics between the reference and reconstructed HRV time series.

## 3. Results 

Table 3 presents the average absolute error (E1) and the average absolute error percentage (E2) of the proposed SpaMA algorithm on all three datasets, respectively. Our SpaMA algorithm is compared to three recently-developed algorithms: TROIKA, JOSS and WFPV. 

The results in Table 3 show that SpaMA has better performance than JOSS and TROIKA for all 12 subjects in the first datasets. In comparison to WFPV, the proposed SpaMA approach outperforms WFPV on average across all 23 subjects in both datasets (1) and (2). The total average of E1 of SpaMA is less than two beats per minutes for all 33 subjects. The average of E1 across the treadmill experiment recordings (activity Type 1- IEEE dataset and Type 4- Chon Lab dataset) is around one beat per minute for all 22 subjects.

**Table 3:** SpaMA algorithm performance comparison.

Subject	Dataset	Activity Type	TROIKA		JOSS		WFPV		SpaMA	
			E1	E2%	E1	E2%	E1	E2%	E1	E2%
1			2.87	2.18	1.33	1.19	1.23	-	**1.23**	**1.14**
2			2.75	2.37	1.75	1.66	1.26	-	1.59	1.30
3			1.91	1.50	1.47	1.27	0.72	-	**0.57**	**0.45**
4			2.25	2.00	1.48	1.41	0.98	-	**0.44**	**0.31**
5			1.69	1.22	0.69	0.51	0.75	-	**0.47**	**0.31**
6			3.16	2.51	1.32	1.09	0.91	-	**0.61**	**0.45**
7			1.72	1.27	0.71	0.54	0.67	-	**0.54**	**0.40**
8			1.83	1.47	0.56	0.47	0.91	-	**0.40**	**0.33**
9			1.58	1.28	0.49	0.41	0.54	-	**0.40**	**0.32**
10			4.00	2.49	3.81	2.43	2.61	-	2.63	1.59
11			1.96	1.29	0.78	0.51	0.94	-	**0.64**	**0.42**
12			3.33	2.30	1.04	0.81	0.98	-	1.20	0.86
mean ± std			2.42 ± 0.8	1.82 ± 0.5	1.28 ± 0.9	1.02 ± 0.6	1.04 ± 0.5	-	**0.89 ± 0.6**	**0.65 ± 0.4**
13	2 (IEEE Cup)	Type (2)					3.58	-	**3.41**	**4.25**
14							9.66	-	**7.29**	**9.80**
15							2.31	-	2.73	2.21
16		Type (3)					4.93	-	**3.18**	**2.11**
17							3.07	-	**3.01**	**2.52**
18							2.67	-	4.46	3.23
19							3.11	-	3.58	3.98
20		Type (2)					2.10	-	**1.94**	**1.66**
21		Type (3)					3.22	-	**2.56**	**2.02**
22							4.35	-	**3.12**	**3.28**
23		Type (2)					0.75	-	1.72	1.97
mean ± std							3.61 ± 2.2	-	**3.36 ± 1.5**	**3.33 ± 2.2**
Type (1,2) mean ± std							2.27 ± 2.0	-	**1.93 ± 2.0**	**2.07 ± 1.7**
24	3 (Chon Lab)	Type (4)							**0.88**	**0.91**
25									**1.03**	**0.83**
26									**1.10**	**0.90**
27									**1.64**	**1.54**
28									**1.41**	**1.12**
29									**0.82**	**0.70**
30									**0.63**	**0.58**
31									**4.78**	**3.87**
32									**0.95**	**0.79**
33									**0.62**	**0.52**
mean ± std									**1.38 ± 1.2**	**1.17 ± 1.0**
Total: mean ± std									**1.86 ± 1.6**	**1.70 ± 1.8**

**Figure 8:**
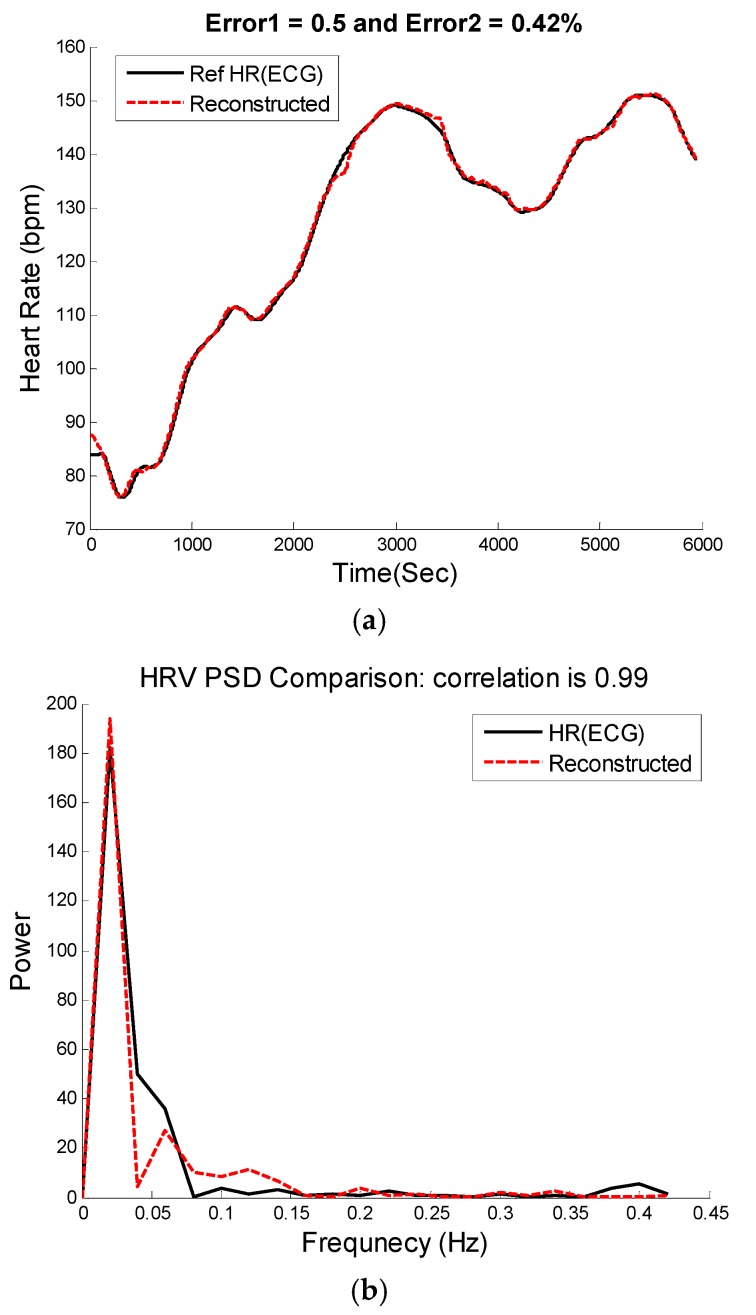
Subject 9 (IEEE Competition Training Dataset). (**a**) Reconstructed HR *vs.* reference HR; (**b**) Spectral comparison of reconstructed HR and reference HR (estimated from reference ECG).

**Figure 9:**
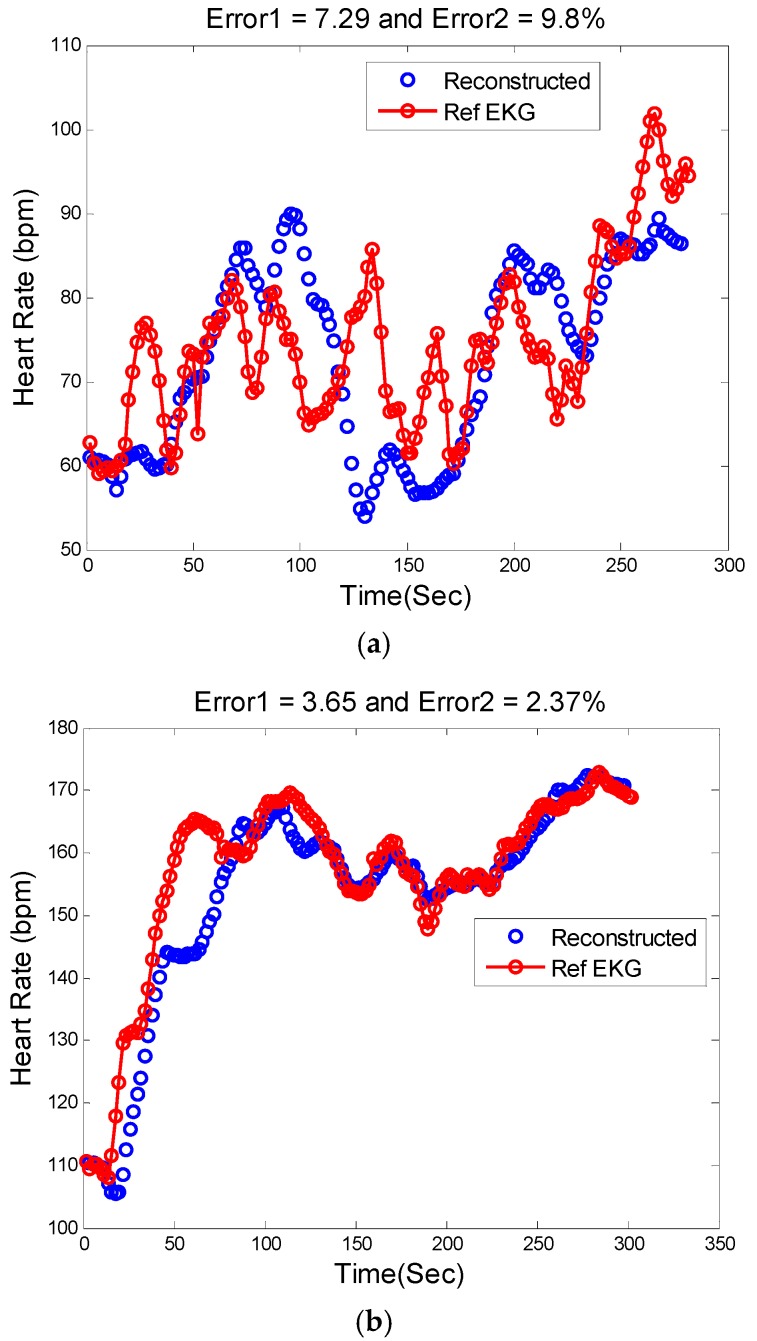
Reconstructed HR *vs.* reference HR: (**a**) Subject 14 (IEEE Competition Test Dataset); (**b**) Subject 16 (IEEE Competition Test Dataset).

**Figure 10:**
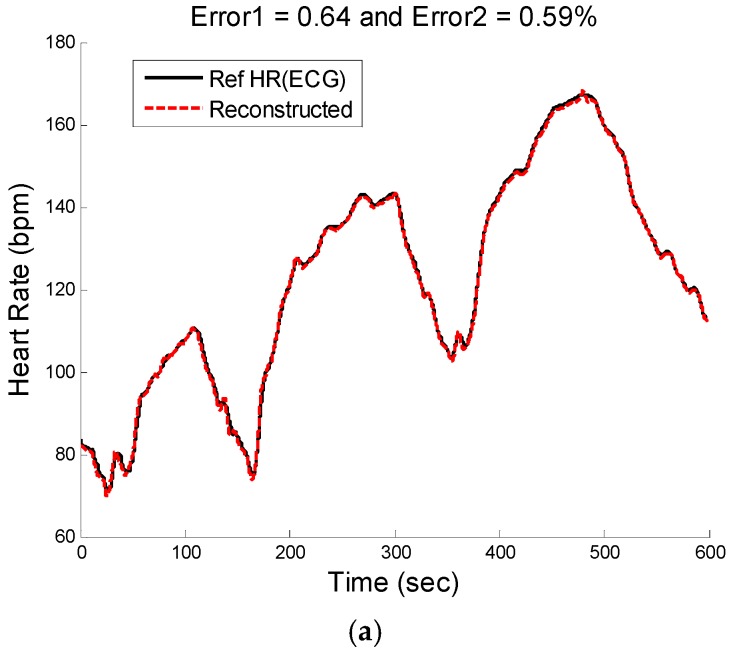

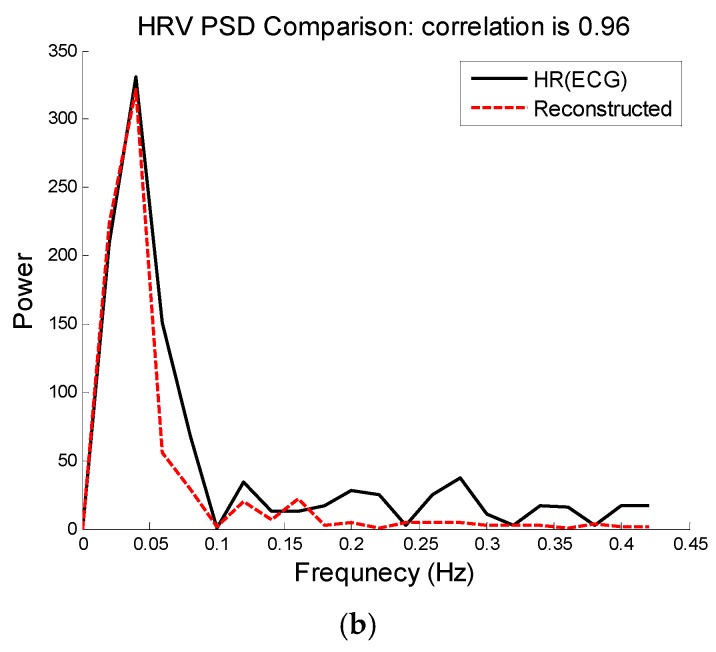
Subject 30 (Chon Lab Dataset). (**a**) Reconstructed HR *vs.* reference HR; (**b**) Spectral comparison of reconstructed HR and reference HR (estimated from reference ECG).

Figure 8, Figure 9 and Figure 10 show the reconstructed HR and corresponding PSD of a sample-sample windowed HR in comparison to the reference HR from ECG. The results for recording #9 from the first dataset (IEEE Competition Training dataset) and activity Type 1 (e.g., running on treadmill) are shown in Figure 8. We can see that the E1 for this particular subject is as low as 0.4 bpm and the correlation between the PSD of reconstructed HR and reference HR is as high as 96%.

Figure 9a illustrates the comparison between the reconstructed HR and the reference HR for subject #14 which has the highest errors. Subject #14 belongs to the second dataset (IEEE Competition Test dataset) and Type 2 activities (e.g., jumping). It can be seen that the largest error is obtained when both the physiological HR and the motion artifacts change rapidly so that the true HR cannot be reliably estimated. Figure 9b shows the comparison results of reconstructed HR and reference HR for recording #16 of the second dataset (IEEE Competition Test dataset) which has the Type 3 activities (e.g., arm exercise).The results for recording #30 from the third datasets (Chon Lab datasets) and activity Type 4 (e.g., running on treadmill) are shown in Figure 10. It can be seen that the E1 for this subject is around 0.6 bpm and the correlation between the PSD of reconstructed HR and reference HR is as high as 99% for LF and 0.96 for HF frequency range. All subjects’ results are provided in Table 4.

**Table 4:** Frequency domain HRV analysis comparison: PSD of SpaMA *vs.* reference.

Subjects	Correlation	
	LF ^1^	HF
1	0.99	0.98
2	0.99	0.96
3	0.99	0.95 *^,2^
4	1.00	0.99
5	1.00	0.99
6	0.99	0.96 *
7	0.98	0.92 *
8	0.97	0.90 *
9	1.00	0.99
10	1.00	0.99
Mean	0.99	0.96

^1^ LF is (0.04-0.15) Hz and HF is (0.15–0.4) Hz; (*) indicates significantly different (*p*-value > 0.05).

Table 4 presents the correlation and statistical difference using the student’s t-test between PSD of estimated and reference HRV in both LF (0.04–0.15 Hz) and HF (0.15–0.4 Hz) frequency ranges. The correlation values in the table are calculated based on Pearson's linear correlation coefficient. As shown in Table 4, there was no difference between the reference and our derived HRV for LF and the difference was seen in only 4 out of 10 subjects for HF. Table 5 shows some of the widely-reported time-domain HRV parameters such as the mean HR, standard-deviation (SDNN) of the normal-to-normal (NN) interval, root-mean-square of successive difference (RMSSD) of the NN interval, and the number of interval differences of successive NN intervals greater than 50 ms divided by the total number of NN intervals (pNN50) estimated from SpaMA in comparison to the reference ECG NN interval. None of these parameters were found to be significantly different between our algorithm-derived and the reference HRV.

**Table 5:** Time domain HRV analysis comparison: SpaMA *vs.* reference HRV.

Subjects	SDNN		meanNN		RMSSD		pNN50	
	SpaMA	Reference	SpaMA	Reference	SpaMA	Reference	SpaMA	Reference
1	2620.75	2566.47	10,481.89	10,480.72	33.24	18.05	0.001	0.020
2	2115.44	2079.58	9908.00	10,020.00	25.93	16.32	0.011	0.019
3	3173.73	3177.68	10,764.20	10,829.06	89.70	56.15	0.019	0.207
4	2517.78	2533.20	10,376.95	10,426.26	13.54	19.58	0.001	0.030
5	2654.42	2670.32	10,846.04	10,990.08	11.88	18.59	0.003	0.018
6	2012.53	1974.65	9737.35	9827.63	39.64	21.17	0.004	0.025
7	3056.36	2925.19	12,519.74	13,134.05	27.66	30.61	0.015	0.071
8	3133.76	2756.66	10,504.00	10,530.00	32.57	36.38	0.002	0.003
9	2195.08	2142.53	10,499.81	10,470.06	8.23	13.01	0.002	0.004
10	2454.57	2406.96	12,936.62	12,981.21	41.52	20.28	0.006	0.024
*p*-value	>0.05		>0.05		>0.05		>0.05	

## 4. Discussion and Conclusions

In this study, a new approach (SpaMA) based on time-varying spectral analysis of the PPG signal is introduced to address heart rate (HR) monitoring in the real world with challenges ranging from a subject who makes sudden movements but is otherwise sedate, to intensive physical activities. The idea behind the proposed SpaMA approach is to compare spectral changes in PPG and accelerometer signals. Three different datasets have been used to verify the algorithm performance. Each dataset reflects different types of activities and movements. In all of the experiments, the reference HR was calculated from an ECG signal that was collected simultaneously with the PPG signal. The estimated HR was calculated from the spectrum of PPG in 8 s time windows. It has been shown in the results section that the proposed SpaMA algorithm can be used for tracking HR changes during severe motion artifacts with an average error of just 1.86 bpm compared to that of the reference ECG; these results are superior to three other algorithms tested: TROIKA, JOSS and WFPV [26,27].

Out of 33 recordings, 23 are from a wrist pulse oximeter, and the rest of the data were recorded by a forehead pulse oximeter. The results from Table 3 show that the SpaMA algorithm can be applied to monitor HR from both wrist and forehead pulse oximeters. By comparing the performance of the algorithm for treadmill experiments (dataset 1 and dataset 3), the error is lower by almost one beat using a wrist pulse oximeter. However, we cannot conclude from this result that the wrist PPG provides less error than the forehead, as the experiments used different subjects and were two separate studies. The prior algorithms (TROIKA, JOSS and WFPV) were tested using only the wrist-based PPG signals as the inventors of these algorithms did not have access to forehead PPG sensors. Our algorithm, tested on data from both PPG sensor locations, proved to be effective regardless of the location of the PPG sensor.

We made several observations while analyzing the data. The tracking ability of the SpaMA algorithm decreased as the frequency changes during recordings increased. This phenomenon mostly was observed while dealing with the second set of datasets from the IEEE competition, which involved Type (2) and Type (3) activities. These types of exercises involved more abrupt movements which consequently made it more difficult to track the HR-related frequencies in the spectrum. In the three datasets that have been analyzed, recordings #10 and 14 are examples of this phenomenon.

The strength of the PPG’s LED is one of the most important factors determining the SpaMA algorithm performance. Movement induces much less spectrum corruption (shift) in the PPG if the LED is sufficiently strong. A reduction in the strength of the PPG signal can also be caused by ambient light leaking into the gap between a PPG sensor and the skin surface [27]. This is because the power of the signal is dependent on the depth and reflection of the light from the pulse oximeter to the subject’s skin. This gap between skin and the planar substrate where the LEDs and PD are mounted may be the result of movement during physical activities or the shape of tissue that the sensors touch. Among the three datasets, low performance for recordings #16 and 31 is the result of a weak PPG signal most likely due to a gap between the sensor and skin caused by motion artifacts.

By using the sample-by-sample windowing process, the proposed SpaMA [33] algorithm can be utilized for both HR monitoring and HRV analysis in both frequency- and time-domains. From the Results section, it can be observed that the algorithm is able to replicate both the low frequency (0.04–0.15 Hz) and the high frequency (0.15–0.4) dynamics well, albeit better the former than the latter, when compared to the reference HRV. For time-domain HRV measures, the mean HR, SDNN, RMSSD, and pNN50 from our algorithm were all found to be not significantly different than the reference HRV. It has long been shown that during dynamic exercise, heart rate increases due to both a parasympathetic withdrawal and an augmented sympathetic activity [34,35]. The relative role of the two drives depends on the exercise intensity [33,36,37]. Analysis of HRV permits insight into this control mechanism [38]. Also, being able to do HRV analysis from PPG even during movement and physical activities would be an advantage for detecting and diagnosing many cardiovascular diseases using only PPG recordings.

The proposed SpaMA algorithm can be potentially implemented in real time. We have found that the algorithm written in Matlab, version R2014 and CPU 3.4 GHz takes only 110 ms per 8 s segments. Therefore, given the high accuracy of the proposed approach in estimating HR despite severe motion artifacts, this method has the potential to be applicable for implementation on wearable devices such as smart watches and PPG-based fitness sensors.
